# In-situ Cutting of Graphene into Short Nanoribbons with Applications to Ni-Zn Batteries

**DOI:** 10.1038/s41598-018-23944-9

**Published:** 2018-04-04

**Authors:** Chao Cui, Mingqiang Li, Xiaoliang Zhang

**Affiliations:** Key Laboratory of Ocean Energy Utilization and Energy Conservation of Ministry of Education, School of Energy and Power Engineering, Dalian University of Technology, Dalian, 116024 China

## Abstract

Rechargeable Ni–Zn batteries, with high safety, low cost and nontoxicity, can be expected to compete with lithium-ion batteries for market share. However, the issue of dissolution of zinc electrode largely limit the battery cycle life and remains unsolved. We designed a kind of graphene-ZnO hybrid electrode in which *in-situ* cutting of graphene into short nanoribbons can effectively anchor plenty of zinc atoms onto the surface of graphene. This not only thoroughly fixes the issue of dissolution of zinc electrode but also increases the specific surface areas of zinc and promotes chemical reaction rate of the charge-discharge processes. By performing experimental measurements, we found that the discharge capacity of the new designed Ni-Zn batteries can be as high as 2603 mAh/g_Zno_, and the superior electrochemical performance can be kept in 10,000 test cycles, suggesting that the new developed *in-situ* cutting technique is very useful in electrochemical fields.

## Introduction

Energy storage has been considered as an important element in modern energy supply chain, which can enhance stability of grids, promote penetration of renewable energy resources, improve energy efficiencies, reduce consumption of fossil energy resources and environmental impacts of energy generation^1^. Among all existing energy storage devices, lithium-ion batteries are becoming more and more popular due to their high energy density, high specific power, and long cycle life^2–5^. However, high cost due to low abundance of lithium and safety issues due to flammable organic electrolyte become the two major bottlenecks for wide promotion of lithium-ion batteries.

Rechargeable Ni–Zn batteries, using aqueous nonflammable solutions as electrolytes, greatly reduces the risk of fire and explosion when batteries are charged or used^6^. Furthermore, natural abundance of zinc largely reduces the cost of Ni–Zn batteries. And compared to toxicity of heavy metals in Ni-Cd batteries and lead-acid batteries, nontoxicity of electrode materials makes Ni–Zn batteries to be environmentally friendly^7^. Moreover, rechargeable Ni–Zn batteries, with high safety and nontoxicity, can be expected to compete with lithium-ion batteries for electric bus. All the above advantages make rechargeable Ni–Zn batteries to be one of promising energy storage systems^6^. However, the low cycle life becomes the obstacle to limit the widespread commercialization of Ni–Zn batteries, which is mainly due to the shape change, dendrite growth, passivation and self-discharge of the zinc electrode^8–14^. In essence, all the above issues affecting the cycle life of Ni–Zn batteries are associated with dissolution of the zinc electrode into solution during charge or discharge processes^15,16^. A variety of attempts have been made to fix the above issues, including adding additives to the zinc electrode or electrolytes^17–24^, changing separators^25–27^, and designing three-dimensional zinc sponge anodes^2^, etc. However, the issue of dissolution of zinc electrode remains unsolved.

Cutting and unzipping technique has been widely used to cut carbon nanotubes into graphene nanoribbons for many applications^28–30^, however, it has been rarely reported to directly cut graphene into short nanoribbons and smaller sized graphene can help to largely improve the mass and specific activity of the oxygen reduction reaction^31^. Here we developed a kind of *in-situ* cutting technique which can directly cut graphene into short nanoribbons, and the strong interatomic interactions between graphene and zinc can help to anchor plenty of zinc atoms onto graphene surfaces. On the one hand, anchoring of zinc atoms onto graphene surfaces can completely solve the issue of dissolution of zinc electrode in alkaline solution during discharge process and avoid dendrite formation; on the other hand, the specific surface areas of zinc can be greatly increased and the chemical reaction rate can be promoted. Furthermore, the superior electrical conductivity of graphene is helpful to largely improve the speed of charge-discharge processes. We performed a series of experiments and molecular dynamics simulations to investigate the electrochemical performance of the new designed rechargeable Ni/Zn secondary battery and the mechanisms behind.

## Results and Discussion

**Figure 1:**
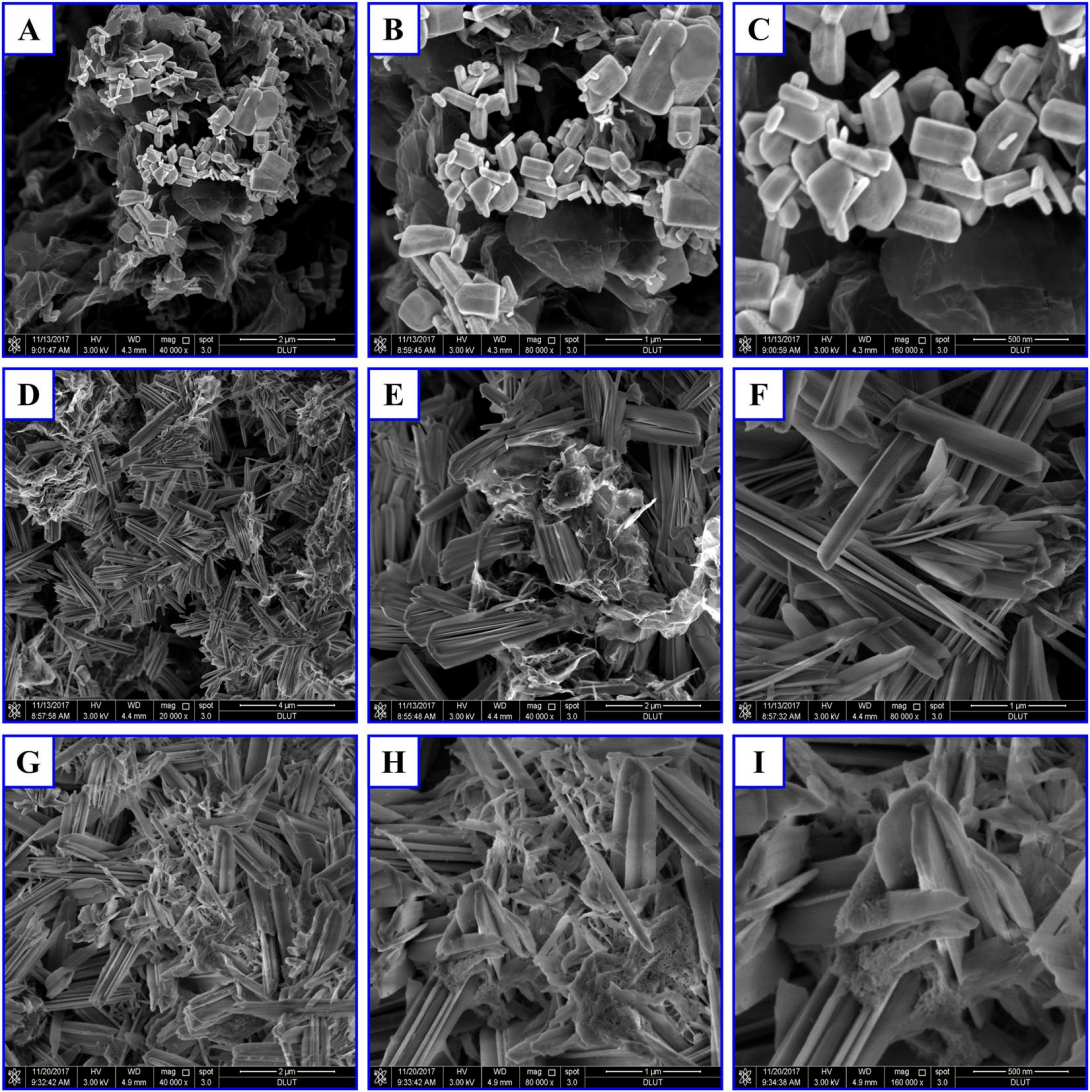
SEM images of the surface morphology of graphene-ZnO hybrid electrode (80.7 wt% ZnO) before cycling (**A**,**B**,**C**) and after 1000 cycles (**D**,**E**,**F**,**G**,**H**,**I**). (**D**,**E**,**F**) and (**G**,**H**,**I**) denote without and with the electrode washed by deionized water.

SEM is used to examine the microstructures of the new designed graphene-ZnO hybrid electrode. The microstructures of the test electrode before cycling are captured by SEM as shown in Fig. 1(A,B,C), from which the hexagonal close packed (HCP) crystal structure of ZnO and large pieces of flexible graphene sheets are clearly identified. After 1,000 charge-discharge cycles in Ni-Zn batteries, the microstructure of the graphene-ZnO hybrid electrode changes significantly as shown in Fig. 1(D,E,F). We can see that the hexagonal close packed (HCP) crystal structure of ZnO and the large pieces of graphene sheets cannot be seen anymore and a kind of new microstructure with the form of several micrometer-long nanosheets emerges in the electrode, which has not been reported in the previous research works. The SEM images suggest that the large pieces of graphene sheets have been cut into short nanoribbons and the Zn atoms formed by the reduction of ZnO crystal is anchored onto the two sides of the graphene nanoribbons, which provides direct evidence to confirm the *in-situ* cutting of graphene. To further confirm that the short nanosheets are not water-soluble materials e.g. KOH, the test electrode (after 1,000 cycles) were washed 12 times with deionized water, and Fig. 1(G,H,I) shows the SEM images of the electrode washed by deionized water, from which we can see that the microstructures remain unchanged suggesting that the short nanosheets are not water-soluble materials.

**Figure 2:**
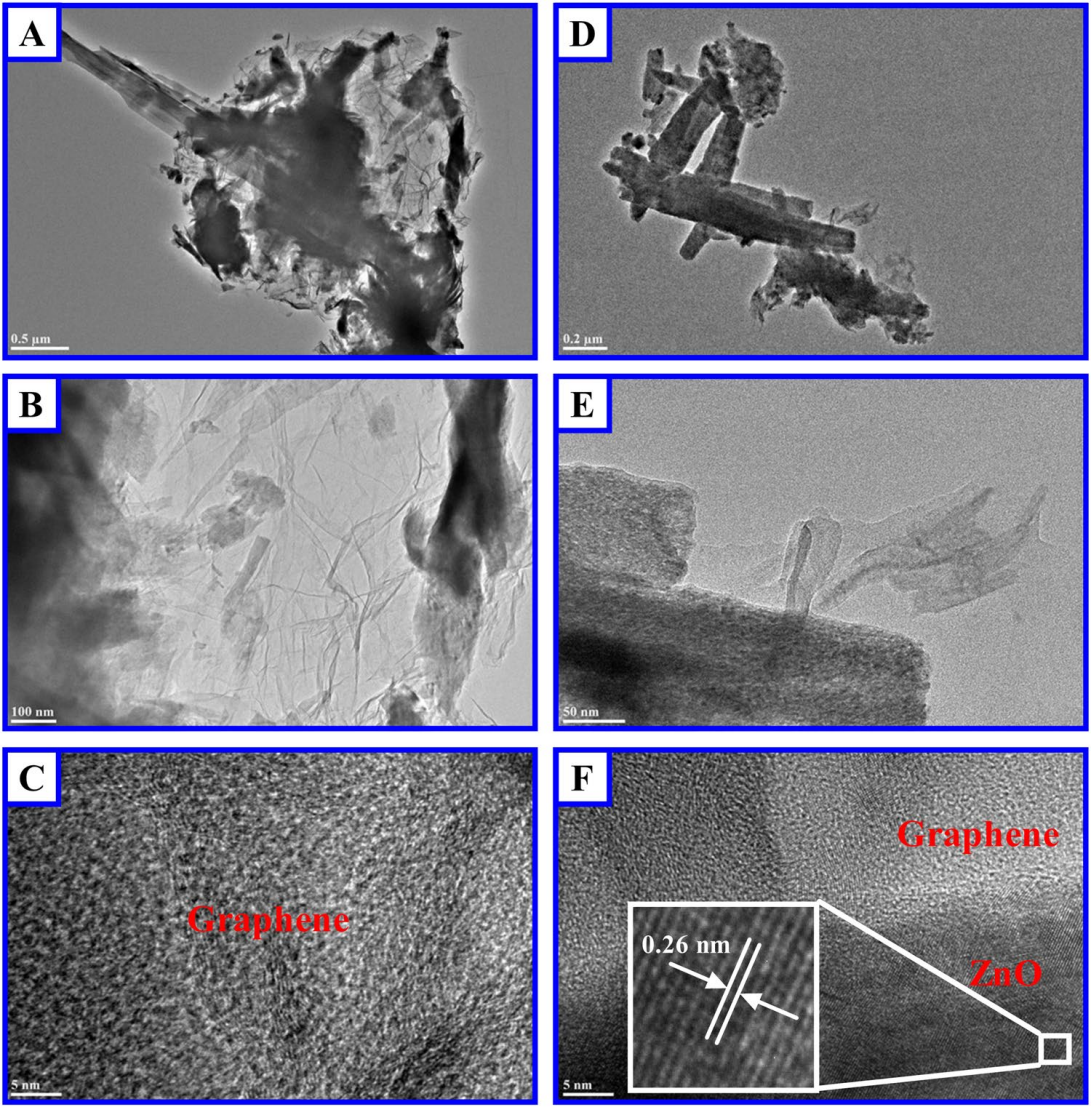
TEM images of the graphene-ZnO hybrid electrode (80.7 wt% ZnO). (**A**,**B**,**C**) Shows the TEM image of pure graphene and (**D**,**E**,**F**) confirms that both graphene and ZnO exist in the electrode.

To achieve a higher resolution to confirm the *in-situ* cutting of graphene, we further performed TEM experiments to investigate the microstructures of the graphene-ZnO electrode. The TEM images of the electrode are shown in Fig. 2(A–C) are used to demonstrate the TEM image of the pure graphene, and we didn’t identify the hexagonal lattice of graphene which should be due to not high enough resolution of the TEM image. Figure 2(D,E) have higher resolution than Fig. 2(A,B,F) has the same resolution as Fig. 2(C), as can be seen in Fig. 2. From Fig. 2(F), the lattice fringes of hexagonal ZnO can be clearly identified with lattice distance of the (002) plane as 0.26 nm. From (D,E,F), we can conclude that both graphene and ZnO exist in the electrode and the *in-situ* cutting of graphene is confirmed.

**Figure 3:**
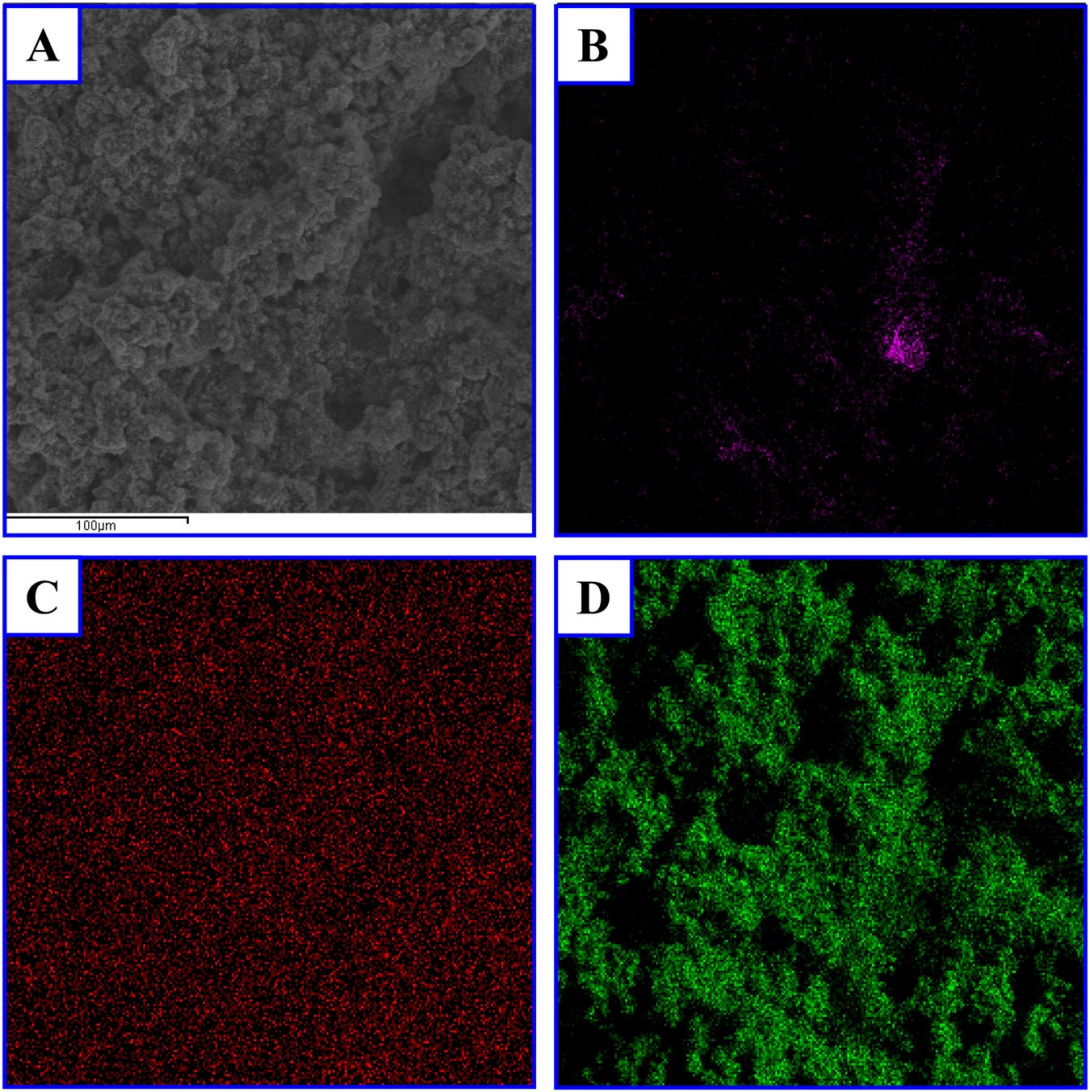
EDS analysis results of the SEM microstructures of graphene-ZnO hybrid electrode (80.7 wt% ZnO) after 1000 cycles. (**A**) SEM image. (**B**) EDS map of C. (**C**) EDS map of Zn. (**D**) EDS map of O.

Energy dispersive X-ray spectroscopy (EDS) analysis was performed to determine the elemental composition of the SEM microstructures in the graphene-ZnO hybrid electrode (80.7 wt% ZnO) after 1,000 cycles and the analysis results are shown in Fig. 3. It can be seen that the Zn, and O elements were dispersed uniformly on the surface of the graphene-ZnO hybrid electrode suggesting a uniform spatial distribution of ZnO in the electrode. However, C elements were dispersed sparsely, suggesting that most of the graphene nanoribbons have been covered by ZnO atoms on their surfaces and cannot be seen in the EDS images.

**Figure 4:**
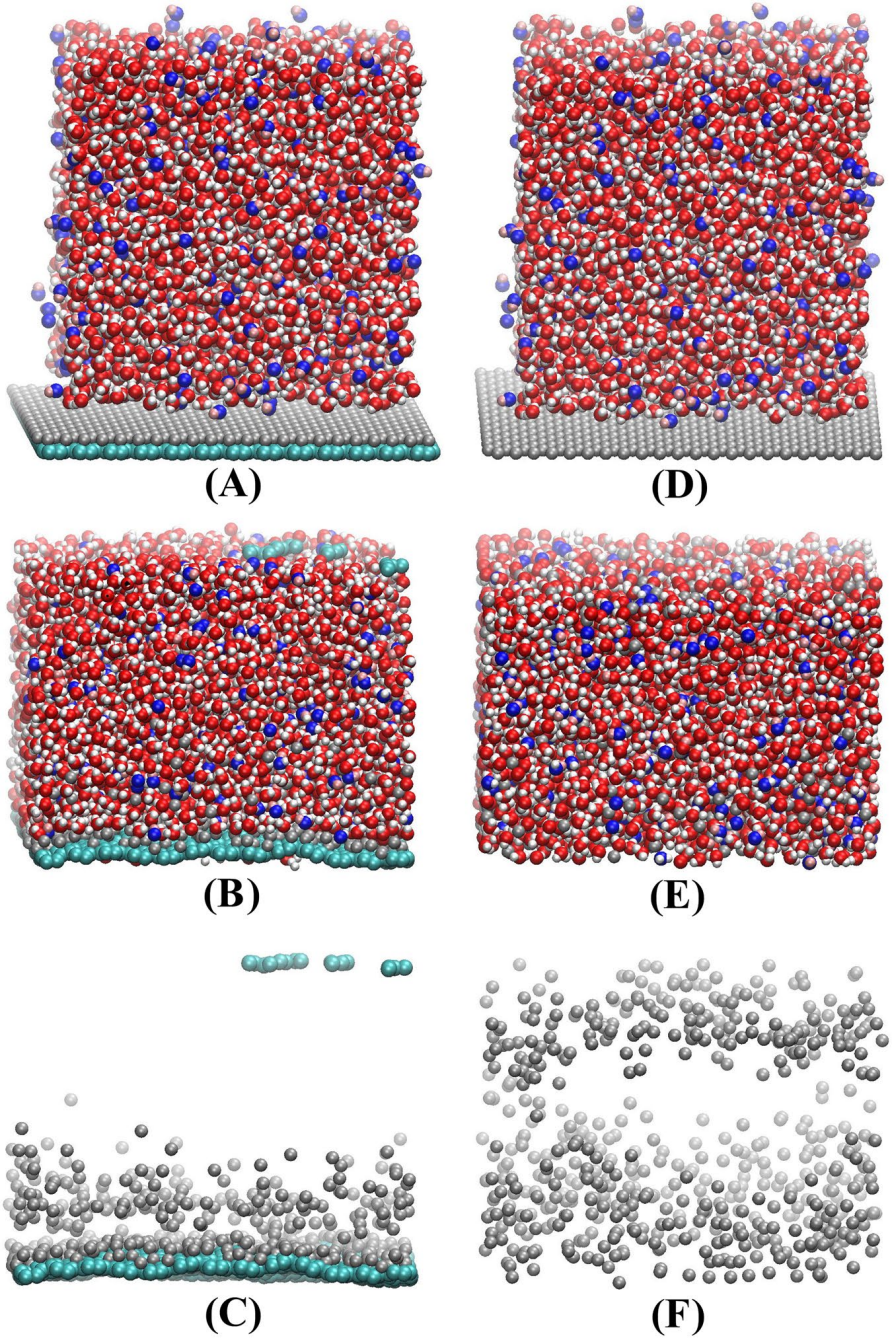
Initial (**A**), final (**B**) structure of the model system of graphene-Zn composite with OH^−^ solution and (**C**) the final structure of graphene and Zn. (**D**), (**E**) and (**F**) are the corresponding structure of the Zn with OH^−^ solution.

To further confirm that graphene can anchor zinc atoms onto its surface, we performed a series of molecular dynamics simulations. All molecular dynamics simulations were performed using the Large-scale Atomic/Molecular Massively Parallel Simulator (LAMMPS) package^32^ with a timestep of 0.1 fs. The reactive force-field (ReaxFF) interatomic potential^33,34^, capable of describing the dynamics of chemical reactions at the atomic scale and widely used to model chemical reactions in batteries^35,36^, was used to describe all the interatomic interactions in the model system. To confirm the key role of graphene to anchor the Zn atoms and avoid their dissolution, we constructed two model systems. One model system consists of 527 OH^−^ ions and 4175 water molecules, corresponding to about 7 mol/L OH^-^ solution which is consistent with our experimental condition, and a single layer of 572 Zn atoms are placed below the OH^−^ solution, and a graphene layer with 1196 C atoms are placed beneath the Zn layer. The size of the whole model system is about 6 nm × 6 nm × 7 nm. To compare with it, the other system with OH^−^ solution and Zn layer the same to the first model system but without graphene layer is constructed. NPT ensemble (constant particles, pressure, and temperature) was used to control the temperature of the model system to be 300 K and the pressure in z direction to be 1 atm. To ensure that the model system achieved the stable status, we set the total running time to be 1,000,000 steps (100 ps). The initial and final structures of the model system of the graphene-Zn composite with OH^−^ solution are shown in Fig. 4(A,B), and for clarity, the final structure of graphene and Zn without OH^−^ solution are shown in Fig. 4(C). After NPT relaxation, we record the number of the Zn atoms within a cutoff distance of 0.5 nm, which reflects the ability of graphene to anchor Zn atoms. In our simulation, 1196 graphene atoms can anchor 738 Zn atoms, corresponding to that 1 g graphene can anchor 3.36 g Zn. Meanwhile the initial and final structures of the model system of the Zn layer with OH^−^ solution are shown in Fig. 4(D,E), and for clarity, the final structure of the Zn layer without OH^−^ solution are shown in Fig. 4(F), suggesting that without graphene, Zn layer will dissolve in the OH^−^ solution which is consistent with experimental observations. By comparison, we can conclude that graphene can play a key role in anchoring Zn atoms and avoid them to be dissolved. Furthermore, from Fig. 4, we can see that the wettability of the electrolyte on the graphene-Zn composite which may influence the performance of the batteries, such as chemical reaction rate, electrode corrosion, etc. Therefore, many researchers designed patterned electrodes to achieve a more lyophilic surface, because surface patterns, e.g., surfaces roughness, play a very important role in solid-liquid interface^37^. From Figs 1 and 3, it can also be seen that the electrode surfaces are not smooth which may influence the performance of the batteries.

**Figure 5:**
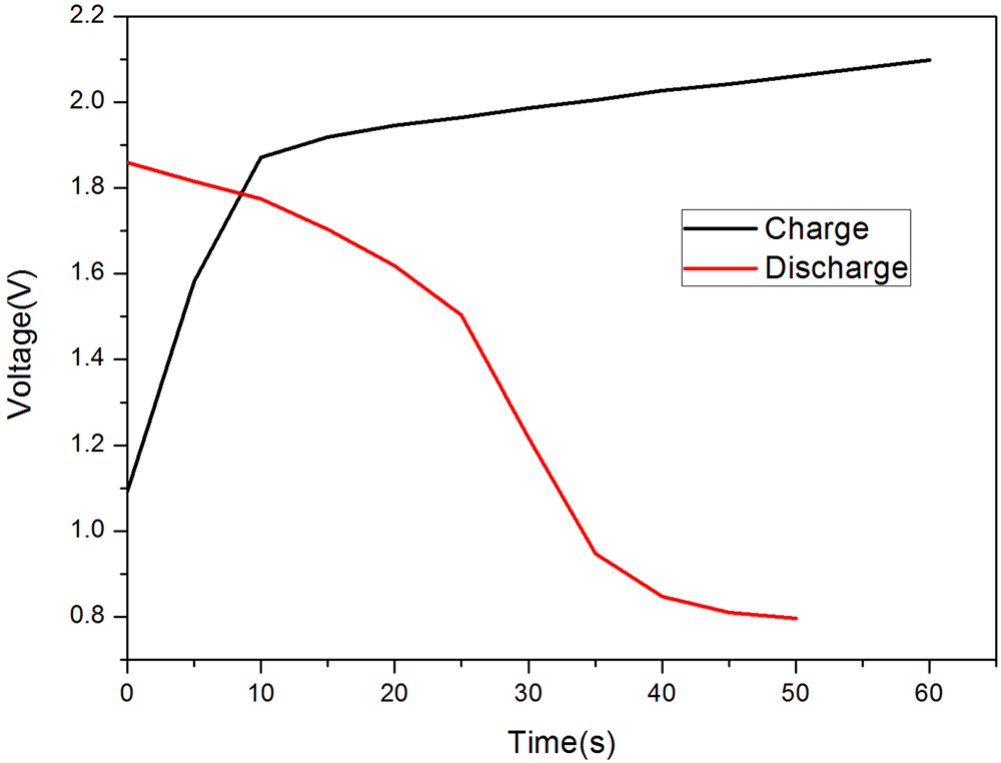
The temporal charge/discharge voltage variation of the battery (cutoff voltage 0.8 V).

To evaluate the cycle life and the stability of the new designed Ni-Zn batteries, we conducted a series of electrochemical measurements in as high as 10,000 test cycles. The temporal change**/**discharge of voltage variation of the battery is shown in Fig. 5. The cutoff voltage is 0.8 V, discharge capacity of ZnO as anode material is about 433.3 mAh/g.

**Figure 6:**
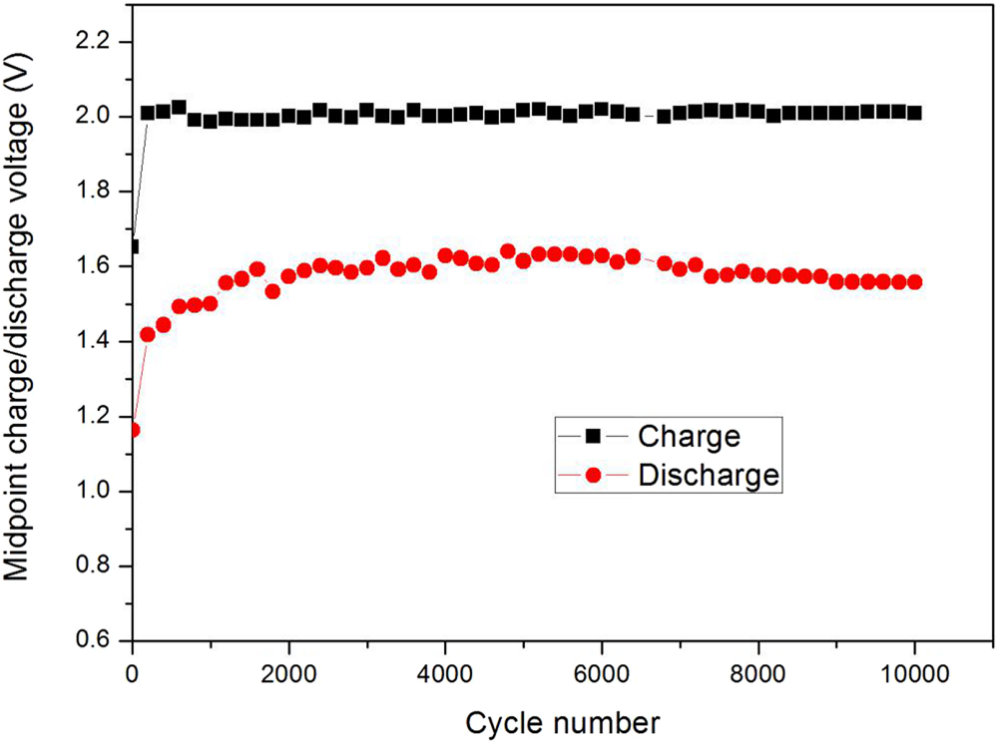
Variation of midpoint charge/discharge voltage vs. cycle number of the graphene-ZnO hybrid electrode (38 wt% ZnO).

**Figure 7:**
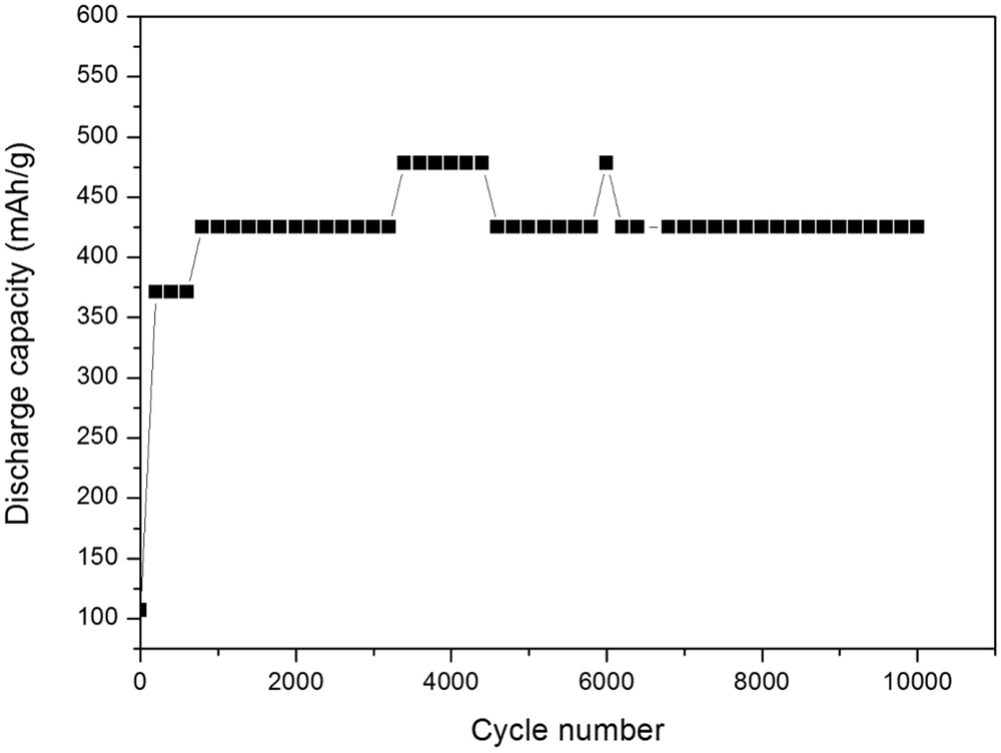
The midpoint discharge capacity of the graphene-ZnO hybrid electrode (38 wt% ZnO).

**Figure 8:**
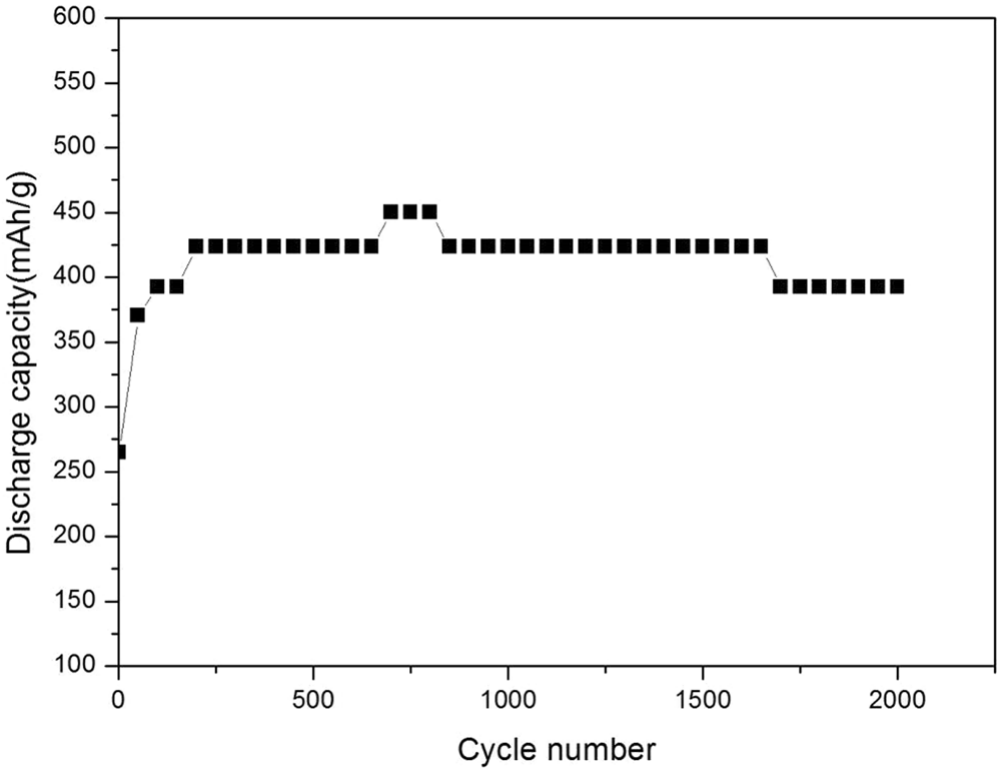
The midpoint discharge capacity of the graphene-ZnO hybrid electrode (80.7 wt% ZnO).

As shown in Figs 6 and 7, the weight percentage of ZnO investigated here is about 38% . At the initial stage, the battery performance is relatively low, but after about 1000 cycles, the battery performance stabilizes at a higher level. This due to the *in-situ* cutting was finished. According to the information shown above, the midpoint charge/discharge voltage is very stable, and the capacity decay rate is only 0.0011% . From the results we can conclude that the electrochemical performance of the new designed Ni-Zn batteries is very stable in 10,000 test cycles and the battery cycle life is far larger than 10,000 cycles. For comparison, we also show the midpoint discharge capacity with the ratio of graphene to zinc oxide of 1:4.19 (80.7% ZnO) (corresponding to the mass ratio of graphene to Zn of 1:3.366 which is close to the critical ratio predicted by the previous molecular dynamics simulations, with this ratio the theoretically minimum quantity of graphene is used) in Fig. 8, and it can be seen that the capacity slightly decreases when the cycle number is larger than 1700, suggesting weaker cycling stability than that in Fig. 7.

**Figure 9:**
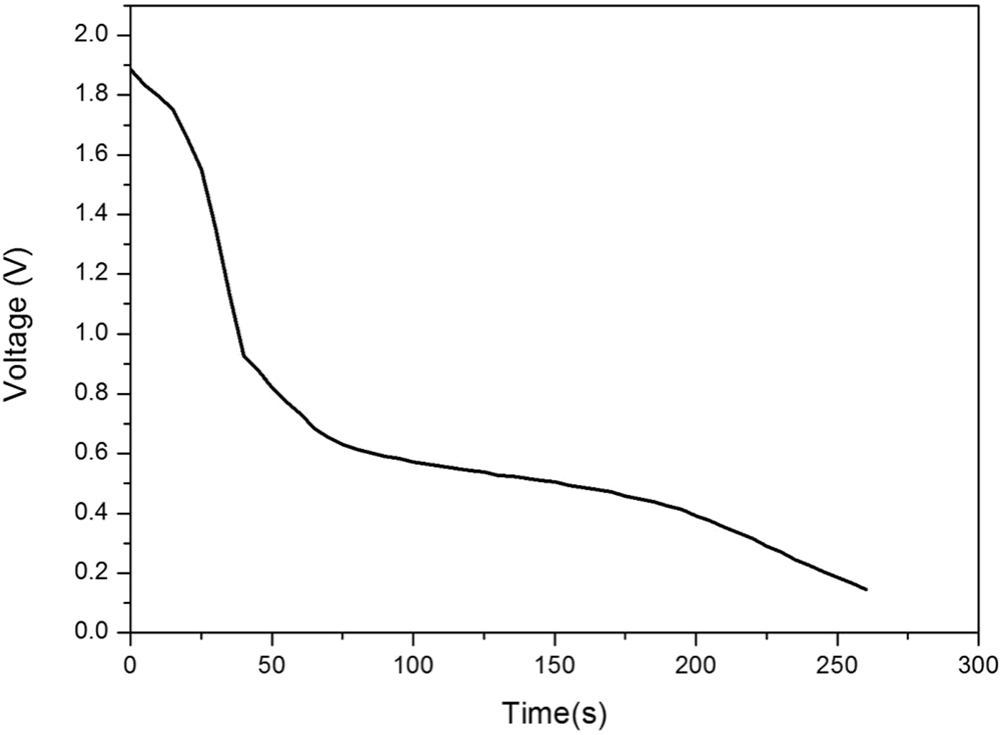
The temporal change of voltage during battery deep discharge process (cutoff voltage 0.2 V).

The temporal change of voltage during deep discharge process of the battery is shown in Fig. 9. When the discharge depth reaches 81.67% (cutoff voltage 0.2 V), discharge capacity of ZnO as anode material is up to 2603 mAh/g_ZnO_, which is far larger than the theoretical specific capacity of Zn electrode of 987 mAh/g_ZnO_^38^. This is far better than the discharge capacity of other ZnO based composite anode materials, in which the discharge capacity cannot maintain beyond 1000 mAh/g_ZnO_. The ZnO–Ni_3_ZnC_0.7_–C composite for lithium ion batteries exhibited an initial discharge capacity of 1120 mAh/g_ZnO_ and retained a discharge capacity of 372 mAh/g_ZnO_ at the 50^th^ cycle^39^. Even though the initial discharge capacity of ZnO-carbon black nanocomposite anode material for lithium ion batteries was up to 2096 mAh/g_ZnO_, which fell quickly to only 1026 mAh/g_ZnO_ after 500 cycles^38^. Reference^40^ constructed a kind of high-power micro-supercapacitor with enhanced capacity that is close to our capacity results by high packing density unidirectional arrays of vertically aligned graphene, so we believe that the superior performance achieved here should be due to the supercapacitor effect of graphene. Furthermore, the properties of the battery, such as cycle life, irreversible capacity loss, self-discharge rate, electrode corrosion and safety are also highly dependent on the thickness of the solid electrolyte interface (SEI) which is a hierarchical structure formed in the transition zone between the electrode and the electrolyte^41^.

## Conclusion

A kind of graphene-ZnO hybrid electrode in rechargeable Ni/Zn secondary battery is designed in which graphene can be *in-situ* cut into short nanoribbons that effectively anchor plenty of zinc atoms onto it surface, possessing largely enhanced cycle life and discharge capacity compared to conventional Ni/Zn secondary battery yet the fabrication method is very easy to operate. The issue of dissolution of zinc electrode is thoroughly solved and the strong interatomic interactions between graphene and zinc atoms and the increased specific surface areas of zinc are the two main mechanisms for the enhanced electrochemical performance.

## Experimental Methods

We synthesized graphene-ZnO hybrid electrode in an easy method: A mixture of graphene and zinc oxide with the mass ratio of graphene to zinc oxide of 1:4.19 (80.7% ZnO) and 31:19 (38% ZnO). The as-prepared ZnO-graphene mixtures were smeared into a brass net (2 cm × 2 cm) to fabricate the anode. Afterwards, the pasted electrodes were dried at 70 °C and pressed at a pressure of 10 MPa. A pasted nickel hydroxide (β-Ni(OH)_2_) electrode is served as the cathode. The ratio of designed capacity for the β-Ni(OH)_2_ cathode and ZnO anode was about 5:1, so the capacity obtained in this present work just reflected the performance of ZnO electrode. A solution of 7 M KOH and 0.1 M LiOH, was used as the electrolyte, and a polyolefin microporous membrane is used as the separator. The ZnO anode and β-Ni(OH)_2_ cathode were assembled into a cell. The microstructure and morphology of the electrodes were characterized by a scanning electron microscope (SEM, Nova, NanoSEM450) and a transmission electron microscope (TEM, JEM-2010).

The galvanostatic charge-discharge tests were conducted on a BS9300 battery program-control test system at room temperature. The cells were charged at 0.1 C for 5 min, and then discharged at 0.1 C down to 0.8 V cut-off. After 1000 test cycles, the *in-situ* cutting was finished.
